# Chlorate of Potassa in Typhoid Fever

**Published:** 1857-03

**Authors:** E. C. Ellet

**Affiliations:** Bunker Hill, Ill.


### ARTICLE III.—*Chlorate of Potassa in Typhoid Fever.* By E. C. Ellet, M.D. Bunker Hill, Ill.

Although not in the habit of writing for the *Journal*, I have, for some time past, been tempted to communicate my views regarding the value of *Chlorate of Potassa* as a remedial agent, and if I can overcome my natural aversion for that kind of notoriety, perhaps I will drop you something on the subject during the winter. Now, I merely wish to draw your particular attention to its value in typhoid fever. For two or three months past that disease has been prevailing rather extensively in this vicinity, and in such a decided form that there is no mistaking its precise nature. I confess I am delighted with the result produced by the free use of the chlorate, and believe it comes nearer what is required in that disease than any other known agent.

I will tell you what first induced me to use it in typhoid fever. Three years ago I read an article in the *American Journal of Medical Sciences*, recommending its use very highly in mercurial salivation. I tried it repeatedly—indeed in every instance yet offered; and can assure you that *so far* it is a true specific. Try it fairly, and you will agree with me. Last Spring my little boy, æt. five, was taken very ill with what I considered infantile remittent fever, as described by writers. As it progressed, growing from bad to worse, I observed his breath growing fetid, with difficulty of swallowing medicine, &c.; no swelling about fauces or submaxillary glands; and, knowing that I had used mercury too cautiously to produce ptyalism, was at a loss for the symptom, until I discovered a long, narrow, ash-colored ulcer situated in the left cheek and horizontaly. At that time his condition was a deplorable one, fever, for some days past, assuming the adynamic type; entire loss of appetite and rapid emaciation. Being already in love with the chlorate, and familiar with Dr. West's opinion of its usefulness in gangrenous sore mouth, &c. I resolved to use it for that symptom particularly—three grains in solution every two hours. After the second dose, the vomiting and all gastric irritability, which from the first had been excessive, ceased—not to return; and although *nothing else* was used after that, his convalescence was rapid without a relapse of any kind. He had been ill eleven days before I began the use of the chlorate; and I, as well as all others, had for two or three days considered his case hopeless.

Subsequently to the convalescence of my child, and before these typhoid cases appeared, I used it in all and every form of sore mouth, whether complicated with febrile symptoms or not; particularly in scorbutic affections, which are quite common in this country according to my experience—thrush, apthæ, nursing sore mouth, spongy gums, foul breath, &c.—with uniform success in all *except* nursing sore mouth, which it will relieve greatly, sometimes cure for a limited period, but will not prevent a relapse—at least not in the way I have used it. Becoming convinced that in some way it exercised wonderful curative power in diseased mucous membranes, particularly those of an ulcerative form, and, knowing from the experience of others that typhoid fever is characterized by that very condition, I resolved to give it a fair trial, and must say that thus far my expectations have been sustained by the result. I have not time now to enter into any further particulars, but if you think what I have said worthy of consideration, will you not try it upon some of your many patients? I should think, from its antiseptic character it would be particularly well adapted for hospital practice.

I have written to you thus familiarly, Dr. Davis, in consequence of my knowledge of your active zeal in the cause that interests us both, and knowing, besides, your superior opportunities for giving any question of this kind a thorough test. I will close by saying that I find the best and most reliable dose for adult in typhoid fever, is twenty grains, every four hours without interruption, so long as febrile symptoms continue, or much sordes continue on the teeth and gums. If fetor of the breath, or offensive dijections exist, you will be astonished to observe how rapidly it disappears. I have had no difficulty about retention of urine in those cases where it has been taken in any size dose over eight grains every four hours. It possesses decided diuretic powers, always causing a free flow of limpid urine. I do not discover that it possesses any *direct* power over hemorrhage from the bowels, neither does it moisten the tongue as turpentine will frequently when prescribed according to your formula. In two or three bad cases, I have prescribed both the turpentine and chlorate alternately for several days in succession, and, as I fancied, with good effect. In a few cases simulating intermittent fever at the beginning, and before I was fully decided regarding the exact nature of the fever, I have kept them on quinine, hydr. cum. cretæ, and turpentine, without observing the least change for the better until I resorted to the use of the chlorate, and discontinued the two first articles, when invariably a change for the better would be rapidly developed.

